# Anti-hyperglycemic Medication Compliance: A Quality Assurance Project

**DOI:** 10.7759/cureus.24421

**Published:** 2022-04-23

**Authors:** Rayan Mamoon, Md Y Mamoon, Debbie Hermanstyne, Issac Sachmechi

**Affiliations:** 1 Endocrinology and Diabetes, Queens Hospital Center, Jamaica, USA; 2 Internal Medicine, Icahn School of Medicine at Mount Sinai, Queens Hospital Center, New York City, USA; 3 Endocrinology and Diabetes, Icahn School of Medicine at Mount Sinai, Queens Hospital Center, New York City, USA

**Keywords:** diabetes self-care, diabetes educational reinforcement, diabetes treatment, insulin regimen, medication adherence strategies

## Abstract

In order to determine the prevalence of adherence among diabetes patients treated at Queens Hospital Center's Diabetes Clinic and to determine barriers preventing adherence, 50 patients were asked a series of questions regarding their medication intake. The majority of patients reported that they understood the self-management steps that were necessary in order to control their diabetes. However, 30% of the interviewed patients with type 1 or type 2 diabetes reported that they missed a dose of their diabetes medication on at least one day in the last month. Forgetting and lifestyle inconveniences were the two most frequently reported reasons for non-adherence. Side effects and problems with the pharmacy or insurance were also significant reasons for non-adherence. Adherence can potentially be increased by combining new forms of treatment and increasing educational reinforcement.

## Introduction

Compliance with medications is arguably the most crucial part in the treatment for diabetes [1]. Diabetes medications are prescribed to perform a variety of essential metabolic functions such as controlling blood glucose levels, stimulating the production of insulin, and regulating the digestion of carbohydrates [2]. Skipping even a few doses each week can increase a patient’s hemoglobin A1c levels and significantly elevate their chances of developing further complications [3,4]. Despite these warnings and constant reminders from their healthcare providers, diabetics often have trouble consistently adhering to their treatment regimens.

Adherence to diabetes treatment is difficult to quantify, but self-report surveys such as the Morisky Medication Adherence Scale survey have been used to predict adherence levels and clinical outcomes in various populations [5]. One multinational study measured adherence by creating a survey for patients and their providers. The study reported that 33.2% of patients skipped their diabetes medication on at least one day during the month of the interview [6]. Similarly, three studies utilizing medication possession ratios observed that 31-46% of patients that were prescribed oral diabetes medications alone or in addition to insulin were non-adherent [5]. A literature review also suggested that the frequency of adherence has been stagnant in recent years and depression along with high costs of medication have been reliable predictors of non-adherence [5]. Another review observed that high levels of education regarding treatment along with strong patient-provider relations are primary indicators of adherence and reduced hemoglobin A1c levels [7]. Our objective was to interview patients in order to assess whether they are routinely complying with their diabetes medications and to identify the reasons for non-adherence.

## Materials and methods

Fifty patients, between the ages of 21 and 81 years (mean age = 56 years), that met with the diabetes educator at Queens Hospital Center’s Diabetes Clinic between July 2020 and July 2021 were asked the following 10 questions regarding their compliance with their medication regimen (Table 1). Located in Queens, NY, the diabetes clinic predominately serves African Americans, Hispanics, and Asian Americans. The Queens Hospital Center Diabetes Self-Management Education Program has been awarded national recognition from the American Diabetes Association four times since 2004 and is recognized as a Diabetes Center of Excellence [8].

**Table 1:** Questions utilized to access the prevalence of adherence to diabetes medications.

Question Number	Question
1	Do you take your diabetes medications regularly?
2	Do you skip your diabetes medications at any time and if yes why so?
3	Can you easily pick up your refills from your pharmacy?
4	Do you understand how and when to administer your diabetes medications?
5	Are you currently taking insulin?
6	If you are taking insulin, do you adjust your insulin dosages depending on your daily diet or activity level?
7	Do you monitor your blood sugar levels?
8	Do you read the directions on your medications?
9	Do you experience any side effects from your diabetes medications?
10	Do you have any suggestions for the diabetes clinic?

The intent of the questions was to determine the prevalence of adherence to diabetes medications, gauge participants’ level of understanding regarding their diabetes medications, and identify any obstacles preventing participants from regularly taking their medications. Gender was not previously determined to affect adherence, so males and females of different ages were selected for this study [9]. Twenty-five male patients and 25 female patients were randomly selected to participate in the study. Interviews were conducted over the phone and any patients who refused to complete the interview or who were not currently prescribed diabetes medications were excluded from the study. The primary questions regarding the frequency of skipping medications were based on a survey used in a previous multinational study that estimated the frequency of skipping diabetes medications [6], and questions regarding accurate administration of insulin were extracted from another international survey-based study [10]. Patients were encouraged to explain their reasons for skipping medications and all responses were instantly transcribed online. Eighty-four percent of the patients interviewed had type 2 diabetes, but patients with type 1 diabetes were also interviewed. The majority of participants were prescribed insulin with at least one more oral medication for diabetes. Answers pertaining to medications for other comorbidities were not included in the results. 

## Results

Table 2 demonstrates how at least 30% of the patients interviewed reportedly skipped their medications on multiple occasions. The occurrences of skipping pertain to instances when patients failed to administer their diabetes medication at times when they were required to take their diabetes medications. Therefore, patients that described a valid medical reason for omitting their diabetes medication at a particular time were not considered to have skipped their medications. Thirty percent of patients that skipped their medications and demonstrated non-adherence may have skipped their oral diabetes medication or may have skipped a dose of their basal insulin. This frequency is consistent with adherence levels measured using the Morisky Medication Adherence Scale surveys and medication possession ratios [5,7].

**Table 2:** Percent distribution of responses to question two.

Response Type	Frequency
Does not skip medications	70%
Reported skipping medications on multiple occasions	30%

The four reasons described in Table 3 prevented 30% of the patients interviewed from fully complying with their diabetes medications. Table 3 indicates that 53.33% of the patients that skipped their diabetes medications forgot to take their medication on multiple occasions, making forgetting the primary reason for skipping diabetes medications. Forty percent of patients that skipped their medications described lifestyle inconveniences such as administering insulin when out with friends and discomfort with injections as a reason for why they skipped their diabetes medications. Side effects such as diarrhea, dizziness, and uncontrollable urination were reported by 20% of patients that skipped their diabetes medications. Consistent with previous studies on reasons for non-adherence to medications, side effects were actually reported by a small percentage of patients that skipped their medications [9]. Problems obtaining new diabetes medications or obtaining refills from the pharmacy were reported by 13.33% of patients that skipped their medications. 

**Table 3:** Frequencies of occurrence for each of the four reasons why patients skipped their diabetes medications.

Reason for Skipping	Frequency of Occurrence (%)
Side effects	20
Problems with the pharmacy/insurance coverage	13.33
Lifestyle inconvenience	40
Forgetting	53.33

According to Table 4, the mean age for patients that skipped their diabetes medication was approximately 62 years, about nine years greater than the mean age for individuals that did not skip their medications. The mean age for individuals that skipped their medications was also about eight years greater than the mean age of the overall cohort. Males constituted the majority of the population that skipped their medications while constituting only 45.71% of the population that did not skip their medications. The percentage of patients with type two diabetes made up 93.33% of the population of patients that skipped their medications. Only 84% of the overall population and 80% of the patients that did not skip their medications had type 2 diabetes; 86% of the overall population was prescribed insulin. The percentage of patients prescribed insulin fell within one percent of 86% in the population that skipped their medication and in the population that did not. Only 53.33% of the patients that skipped their medications regularly monitor their blood sugar levels. Further, 71.43% of the patients who do not skip their medications and 64% of the overall cohort claimed to regularly monitor their blood sugar levels; 26.67% of the population that skipped their medications were dependent on caregivers for directly administering their medications or managing their medication intake schedule. Patients that fell into this category claimed that visual impairments, forgetfulness, or other disabilities prevented them from remembering to take their medications, administering Baqsimi during a hypoglycemic emergency, or monitoring their blood sugar. Only 10% of the overall population and 2.86% of the population that did not skip their medications required this form of medication assistance. Ninety-two percent of the overall population claimed that they understood the importance of their medication regimen and understood the instructions given to them regarding their diabetes medications. All 35 patients that did not skip their medications claimed to have this knowledge while only 73.33% of the patients that skipped their medication claimed to have complete knowledge regarding their diabetes medications.

**Table 4:** Demographic analysis of overall cohort, population that skipped their diabetes medication, and population that did not skip their diabetes medications.

Demographic	Did Not Skip Medications (N=35)	Skips Medications (N=15)	Overall Cohort (N=50)
Mean age (SD) years	53.42857 (15.46778)	62.13333 (9.29567)	56.04 (14.24073)
Male, N (%)	16 (45.71%)	9 (60.00%)	25 (50.00%)
Type 2 diabetes mellitus, N (%)	28 (80.00%)	14 (93.33%)	42 (84.00%)
Taking insulin, N (%)	30 (85.71%)	13 (86.67%)	43 (86.00%)
Monitors blood sugar, N (%)	24 (71.43%)	8 (53.33%)	32 (64.00%)
Required assistance administering medications, N (%)	1 (2.86%)	4 (26.67%)	5 (10.00%)
Claimed to understand instructions, N (%)	35 (100.00%)	11 (73.33%)	46 (92.00%)

## Discussion

Ninety-two percent of the interviewed patients reported that they understand how to properly administer their diabetes medications. Seventy percent of patients reported that they did not skip their medications at any time, indicating that the clinic is doing a thorough job at educating their patients and motivating them to comply with their medications. The other 30% of patients reported skipping their medication anywhere from once a month to multiple times a day. There were four reasons why patients claimed to skip their medications and nearly one-third of the patients had multiple reasons for skipping. 

The most frequent reason for why patients skipped their medication was because they would forget to take their medication at the correct time. Two individuals over the age of 60 years who forget to take their medications asked for pill boxes so that they would have an easier time organizing their medications. Another patient who forgot to take her Novolog dose before a meal took it after her meal, but experienced hypoglycemia. Forgetting is the simplest explanation for why people skip their medications, but it is also arguably the hardest issue to overcome. The mean age for patients that skipped their medication was about 63 years, and it might be inevitable for some patients to forget to administer their diabetes medications. Reinforcing how patients should adjust their dosages when they forget to take their medications can be crucial, in preventing patients from experiencing hypoglycemia or hyperglycemia [11]. Setting alarms in phones and implementing new smart insulin pens such as Inpen could be beneficial for reminding patients to administer their medications consistently [12].

Inconvenience to lifestyle was the second most frequent reason for why patients skipped their medications. Taking insulin before lunch specifically seems inconvenient for patients. Another study also observed consistent administration of the morning dose and irregular consumption of the lunch time dose [13]. One patient admitted that he skips his lunchtime insulin when he goes out and compensates by taking insulin at two times that he was not instructed to do so. Social pressures seem to take a role in forcing patients to defy the instructions given to them by their clinician. Combining dose drugs may make it more convenient for patients to adhere to their medications, but one study noticed a significantly greater rate of overconsumption among patients prescribed one daily dose compared to patients prescribed three daily doses [13]. Since the percentage of patients prescribed insulin was approximately the same for patients that skipped their medication and for patients that did not, patients prescribed insulin were equally as likely to skip their medication as patients prescribed only oral medications. Fear of needles is another inconvenience for patients prescribed insulin, but this problem can be alleviated using microneedles that are thinner than hair and not painful [14].

The third reason, avoidance due to persistent side effects was also a significant reason that caused some patients to skip their medication. Twenty percent of the patients that do not currently skip their medications previously discussed side effects or lifestyle inconveniences with their clinicians and had their regimens quickly adjusted so that they no longer experienced those effects. It’s likely that clinicians can effectively provide alternatives for most patients who are experiencing side effects, but one patient who skips her medication claimed that she does not always remember to tell her clinicians all her problems. Patients should be encouraged to write down any problems with side effects or lifestyle inconveniences they may encounter with their medication regimen. Advanced electronic monitoring that actively monitor patients’ blood sugar levels and how often they administer their medications may also help providers more accurately record the frequency of medication non-adherence [15]. The fourth reason for non-adherence with medication is issues with the pharmacy and insurance coverage. Problems with obtaining medication involved delays in pharmacies receiving updated prescriptions for medication or for blood glucose testing strips. 

Further, 73.33% of the patients that skipped their medication claimed that they did not understand the instructions given to them at the diabetes clinic or they do not understand the importance of staying adherent to their medication regimen. This may entail that the patient forgot the instructions given to them by the diabetes educator and the healthcare providers at the clinic or they may disagree with the healthcare providers on the necessity of their current diabetes medications. Since 26.67% of the patients that skipped their diabetes medication required assistance administering their diabetes medication, involving family members, friends or home health aides in the treatment plan will likely help dependent patients remember to take their medications and become more motivated to consistently administer their diabetes medications. For example, none of the five patients who had family members present with them during their interviews skipped their medications. Switching from daily basal insulin injections to once weekly basal insulin injections such as Icodec may also improve adherence for patients that require medication assistance [16]. 

Some have questioned the credibility of surveys in accurately identifying the rate of adherence, but one meta-analysis observed a significant correlation between the results of self-reported surveys and the results of medication possession ratio studies, suggesting that surveys are reliable predictors of adherence [17]. Another study of similar size utilized surveys to measure medication adherence in another minority population of diabetes patients and identified a 56% frequency of nonadherence [18]. The prevalence of adherence likely varies greatly with location. Nevertheless, forgetting and problems obtaining medications were also common reasons for nonadherence [18]. Increasing adherence to medications can be vital in improving quality of life for many patients and increasing the likelihood that they will follow other instructions related to their treatment plan [7].

## Conclusions

Patients who were interviewed in this study mostly demonstrated a high degree of adherence to their medication regimen. Forgetting and inconveniences to lifestyle are the two most common reasons why patients in this study reportedly skipped their medications. The other two reasons, side effects and problems with the pharmacy or insurance, were much less common. Despite the best efforts of the patients and the medical staff at the diabetes clinic, 30% of patients reportedly skipped their diabetes medications. The frequency of nonadherence is similar to the 33.2% frequency of skipping that was measured in a multinational diabetes medicine adherence study conducted in 2012. The prevalence of regular monitoring of glucose levels was 18.1% higher in patients that did not skip their medications compared to patients that skipped their medications, suggesting that self-monitoring and education are crucial predictors of adherence to medications. Overcoming forgetfulness and lifestyle inconvenience, the two biggest causes of non-adherence in this study, may require advanced forms of treatment such as smart insulin pens or once-weekly insulin doses which are less burdensome. For the elderly and socioeconomically diverse population served in Queens Hospital Center's Diabetes Clinic accessibility of these novel forms of treatment may be even more impactful.
